# Is the routine drainage after surgery for thyroid necessary? - A prospective randomized clinical study [ISRCTN63623153]

**DOI:** 10.1186/1471-2482-5-11

**Published:** 2005-05-19

**Authors:** Jotinder Khanna, RS Mohil, Chintamani, Dinesh Bhatnagar, MK Mittal, M Sahoo, Magan Mehrotra

**Affiliations:** 1 Department of Surgery, Institute of Nuclear Medicine and Allied Sciences, Delhi, India; 2 Department of Cytopathology, Institute of Nuclear Medicine and Allied Sciences, Delhi, India; 3 Vardhman Mahavir Medical College Safdarjang Hospital, New Delhi-India; 4 Department of Radiology, Institute of Nuclear Medicine and Allied Sciences, Delhi, India

## Abstract

**Background:**

Drains are usually left after thyroid surgery to prevent formation of hematoma and seroma in the thyroid bed. This is done to reduce complications and hospital stay. Objective evaluation of the amount collected in the thyroid bed by ultrasonography (USG) can help in assessing the role of drains.

**Methods:**

A randomized prospective control study was conducted on 94 patients undergoing 102 thyroid surgeries, over a period of fifteen months. Patients included in the study were randomly allocated to drain and non-drain group on the basis of computer generated random number table. The surgeon was informed of the group just before the closure of the wound Postoperatively USG neck was done on first and seventh postoperative day by the same ultrasonologist each time. Any swelling, change in voice, tetany and tingling sensation were also recorded. The data was analyzed using two-sample t-test for calculating unequal variance.

**Results:**

Both groups were evenly balanced according to age, sex, and size of tumor, type of procedure performed and histopathological diagnosis. There was no significant difference in collection of thyroid bed assessed by USG on D1 & D7 in the two groups (p = 0.313) but the hospital stay was significantly reduced in the non-drain group (p = 0.007). One patient in the drain group required needle aspiration for collection in thyroid bed. No patient in either group required re-operation for bleeding or haematoma.

**Conclusion:**

Routine drainage of thyroid bed following thyroid surgery may not be necessary. Not draining the wound results in lesser morbidity and decreased hospital stay.

## Background

Most surgeons give into tradition of leaving a drain following thyroid surgery with the hope that this will obliterate the dead space and evacuate collected blood and serum. This belief is further reinforced by the fact that postoperative drains usually yield fluid. The need for drainage has been questioned after various types of surgeries with much larger potential dead spaces like cholecystectomy and colonic anastamosis [1,2]. These procedures are now routinely not drained. Blood and serum that they are supposed to drain usually block drains. They add to discomfort, give extra scar and increase hospital stay. We carried out a prospective randomized study to study the role of drains after thyroid surgery and monitor the fluid collection in thyroid bed objectively by USG.

## Methods

The approval was taken from the reviewer board and ethical committee of the hospital before initiating the study and informed consent was taken from the patients regarding the use of drains. The study was carried out on 94 patients who underwent 102 thyroid surgeries in a single unit between January 2001 and March 2002. This discrepancy was due to 8 patients undergoing completion thyroidectomies for well differentiated thyroid carcinomas confirmed by histopathology.. These 8 patients were randomized as fresh cases with no consideration given to previous surgery. Patients with cervical lymph nodes metastases requiring neck dissection and those with clinical or laboratory indicators of coagulation disorders were excluded from the study. No patient was excluded on the basis of size of the gland, difficulty in surgery, surgery involving both lobes and re-operation in the neck. Alll patients underwent routine preoperative and postoperative laryngoscopy(indirect/direct) as a part of the protocol at our center. The patients were randomly allocated to drain and non-drain group on the basis of computer generated random number table. The operating surgeon was informed of the group just before the closure of the wound. In the drain group a closed suction drain with negative pressure (Romovac^®^) was brought out through a separate wound. Ultra sound of the neck using B mode with linear frequency of 7.5 MHz was performed in both groups between 24 – 48 hours of surgery and seventh postoperative day each time by the same radiologist or under his supervision. Volume of fluid collection in the operative bed was calculated by measuring the maximum diameter in three dimensions. The volume of fluid collected in the suction drain was measured separately. The drains were removed in all the patients after 48 hours. Before the present study was contemplated, a pilot study was carried out on 20 patients(excluded from the present study)to ascertain the duration of drainage and the drains were removed after the drainage reduced to less than 30 ml in 24 hours following which the patients were discharged. It was observed that the average time taken for the drains to be removed was 4 days while the ultrasound assessment did not reveal any collection in the thyroid bed after 48 hours. The review committee of the hospital therefore recommended keeping of the drains for 48 hours in order to compare the morbidity in the two groups. Therefore a cut off period of 48 hours for removal of drains was considered. All patients were observed for any postoperative respiratory distress, change in voice, wound collection, tingling sensation and tetany. The specimens were subjected to histopathological examination for final confirmation of diagnosis. Using two sample "t" test all the data was statistically analyzed for any significant difference in the two groups for a) fluid collection in thyroid bed on day one and day seven, b) size of nodule and amount of fluid collection and c) complication rate.

## Results

The average age of patients in the present study was 34.56 years (range 8–60 years). Male to female ratio was 1: 6.84, with equal distribution in both the groups based on the type of surgery and size of nodule. The amount of fluid collection in thyroid bed as assessed by USG for both the groups on day one and day seven is shown in Table 1. Two-sample T- test was applied for detecting any difference in means of fluid collected between the two groups. There was no statistically significant difference in the volume of fluid collection on day one (p = 0.371) and day seven (p = 0.577) between the two groups. On the other hand the amount of fluid collected in the suction drain was noted for 48 hours. The average collection was 167.14 ml (range 30 – 120 ml/ day).

**Table 1:** Volume of fluid collection in the two groups as assessed by USG on D 1 & D7

Amount of fluid	Drain group		Non drain group	
	
	D1	D7	D1	D7
Mean	3.258 ml	1.819 ml	2.345 ml	1.366 ml
Minimum	0 ml	0 ml	0 ml	0 ml
Maximum	40 ml	35 ml	19 ml	14.2 ml
Total number of patients	51	51	51	51
Two sample T test for detection of difference in means of fluid collected on D1 & DD7 respectively				
Unequal variance	T	DF	P	
D1	0.9	81.5	0.371	
D7	0.58	72.9	0.577	

Average size of thyroid nodule was 4.03 cm & 4.12 cm in the drain and non-drain groups respectively. Kruskal – Wallis one way non parametric analysis showed no significant statistical difference in the amount of collection according to size of nodule arbitrarily taken as < 4 cm & > 4 cm on D1 & D7 respectively (Table 2 &3).

**Table 2:** Chi Squared approximation for difference in volume of collection based on size on D1

Amount of fluid in ml	Drain group		Non drain group	
	
	<4 cm	>4 cm	< 4 cm	>4 cm
Mean D1	2.30 ml	5.935 ml	2.452 ml	2.011
Total number of patients	37	14	34	17

P = 0.313 (ns)

**Table 3:** Chi Squared approximation for difference in volume of collection based on size on D7

Amount of fluid	Drain group		Non drain group	
	
	<4 cm	>4 cm	< 4 cm	>4 cm
Mean	0.956 ml	1.956 ml	1.388 ml	1.244 ml
Total number of patients	37	14	34	17

P = 0.0712 (ns)

Average duration of hospital stay was 3.715 days for the entire group; 4.35 days for drain group and 3.07 days for non-drain group. This was statistically significant when analysed using two-sample T test (P = 0.0072).

Complications that were observed in the present study are shown in Table 4. Of the six patients who developed collection, four had undergone bilateral subtotal thyroidectomy with an average amount 13.40 ml. Only one patient from the drain group required single aspiration. None of the patients had respiratory distress. Three patients had tingling sensation and two patients developed transient tetany. Five patients developed transient change in voice four of these belonged to drain group, the difference being non significant (p= 0.36). One patient in each group developed wound infection. The presence or absence of drains, expectedly did not contribute significantly to the postoperative complications.

**Table 4:** Distribution of complications into drain and non-drain group

Complication	Drain group	Non drain group
Swelling	3	3
Tetany	0	2
Tingling	1	2
Infection	1	1

## Discussion

Drains have been traditionally used in most of the surgical procedures with limited evidence to suggest any benefit [1-4]. The present prospective randomized study has failed to show any advantage of routinely using drain after uncomplicated thyroid surgery. Except for patients undergoing simultaneous neck dissection or coagulation disorder no other exception was made based on factors mentioned above.

Other authors in randomized studies reported similar results with sample size varying from 100 to 200 patients (5 -10). Two large non-randomized studies of 250 and 400 patients have also documented no benefit of using drains after thyroid surgery [11,12]. Objective assessment of fluid collection in thyroid bed by USG has been done very infrequently in randomized settings. Debry et al have used it selectively in two patients developing postoperative haematoma while Schwartz used it to compare two types of drains [5,13]. In the present study absence of fluid in the thyroid bed on USG but its presence in the suction drain could therefore be due to the drain itself [8]. The drains by virtue of the inflammation induced due to their presence may actually increase the drainage. The vaccum created by the negative suction of the drain may prevent the lymphatics from sealing off and thus cause increase in the seroma formation and drainage [5-9].

## Conclusion

The present randomized study highlights that placement of drains after routine thyroid surgery may induce rather than prevent fluid collection, is not related to the type of surgery or size of nodule, has no influence on complications, leads to an extra scar and may increase the hospital stay (if the patients can not be discharged with drains in situ). Meticulous haemostasis and attention to finer details during surgery are more important. Routine use of drains after thyroid surgery may therefore not be necessary.

## Competing interests

The author(s) declare that they have no competing interests.

## Authors' contributions

DB was the chief surgeon in charge of the unit, the department of surgery also responsible for the designing of the study, RSM, CM were the senior operating surgeons, JK, MM were the senior residents in charge of the cases did the statistical analysis, MK did the ultrasonographic assessment and MS was responsible for the pathological analysis. All authors read and approved the manuscript.

## Acknowledgements

We would like to acknowledge with gratitude the contribution of Dr Rajvir Singh PhD (Biostatistics) Statistician All India Institute Of Medical sciences for the statistical analysis.

## Pre-publication history

The pre-publication history for this paper can be accessed here:


